# Myeloid-derived suppressor cells in hematologic malignancies: two sides of the same coin

**DOI:** 10.1186/s40164-022-00296-9

**Published:** 2022-07-19

**Authors:** Shunjie Yu, Xiaotong Ren, Lijuan Li

**Affiliations:** Department of Hematology, Tianjin Medical University General Hospital, Heping district 154 Anshan Road, Tianjin, China

**Keywords:** Hematologic malignancies, Myeloid-derived suppressor cells, Immunotherapy

## Abstract

Myeloid-derived suppressor cells (MDSCs) are a heterogeneous population of bone marrow cells originating from immature myeloid cells. They exert potent immunosuppressive activity and are closely associated with the development of various diseases such as malignancies, infections, and inflammation. In malignant tumors, MDSCs, one of the most dominant cellular components comprising the tumor microenvironment, play a crucial role in tumor growth, drug resistance, recurrence, and immune escape. Although the role of MDSCs in solid tumors is currently being extensively studied, little is known about their role in hematologic malignancies. In this review, we comprehensively summarized and reviewed the different roles of MDSCs in hematologic malignancies and hematopoietic stem cell transplantation, and finally discussed current targeted therapeutic strategies.Affiliation: Kindly check and confirm the processed affiliations are correct. Amend if any.correct

## Introduction

Since the discovery of myeloid-derived suppressor cells (MDSCs) in 2007 [1], scientists have been studying their biological properties, as well as their physiological and pathological roles, with increasing fervor. MDSCs are composed of two main classes of cells, namely monocytic MDSCs (M-MDSCs) and granulocytes or polymorphonuclear MDSCs (PMN-MDSCs). The former is phenotypically and morphologically similar to monocytes, whereas the latter is closer to neutrophils [2]. A minority of bone marrow progenitor cells and precursor cells with colony-forming activity in humans, referred to as early MDSCs, has also been discovered in recent years (e-MDSCs) [3]. In pathological conditions such as infections, malignancies, and chronic inflammation, multiple growth factors and inflammatory mediators stimulate MDSCs to undergo expansion, further participating in immune regulation and disease development [4–6].

In patients with solid tumors, the number of MDSCs is positively correlated with cancer stage and tumor load [11, 12] and is a biological marker of treatment failure as well as poor prognosis [13–15]. Under the joint regulation of the tumor microenvironment and tumor-derived factors secreted by cancer cells, MDSCs can be amplified and recruited to tumor primary and metastatic sites [16, 17], while activated MDSCs can suppress the anti-tumor immune response of immune cells (e.g., NK cells, CD4^+^ T cells, and CD8^+^ T cells) through numerous mechanisms (e.g., the release of nitric oxide or reactive oxygen species [7], depletion of essential metabolites [8], induction of other immunosuppressive cells [9], and expression of negative immune checkpoint molecules [10]) in the tumor microenvironment, thus promoting tumor cells immune tolerance and leading to their immune escape. Moreover, MDSCs can exacerbate immune dysfunction in the tumor microenvironment and form a feedback loop to further enhance their own accumulation and expansion by secreting pro-inflammatory proteins such as S100A8/9 [18, 19]. Therefore, targeted therapies against MDSCs to restore the effective anti-tumor response capacity of immune cells have emerged as an important goal in tumor therapy. However, although MDSCs can promote tumor progression and immunosuppression in hematologic malignancies, they do not exclusively have negative effects [20, 21], especially in allogeneic hematopoietic stem cell transplantation (allo-HSCT) for the treatment of hematologic malignancies, where MDSCs play a positive role between graft-versus-leukemia (GVL) effects and host immune tolerance [22, 23].

In this review, we first described the main characteristics of MDSCs and their immunosuppressive functions. Next, we elaborated on the role of MDSCs in hematologic malignancies such as lymphoma, multiple myeloma, leukemia, and hematopoietic stem cell transplantation. Finally, we discussed and summarized the potential of targeting MDSCs as a treatment strategy for malignant hematologic diseases.

### Phenotypic characteristics of MDSCs

MDSCs are defined as a heterogeneous population of bone marrow cells with potent immunosuppressive activity originating from immature myeloid common progenitor cells (CMP). They can be divided into two subpopulations in mice, M-MDSCs (CD11b^+^ Ly6C^hi^ Ly6G^-^) and PMN-MDSCs (CD11b^+^ Ly6C^lo^ Ly6G^+^ cells). In humans, the M-MDSCs and PMN-MDSCs (G-MDSC) subpopulations are labeled as Lin^-^ (CD3, CD19, CD56) CD11b^+^ CD15^-^ CD14^+^ HLA^-^ DR ^lo/-^ and Lin^-^ CD11b^+^ CD15^+^ CD14^-^ CD66b^+^ HLA-DR ^lo/-^, respectively. In humans, there are also immature and early MDSCs (e-MDSC) subpopulation labeled as Lin^-^ HLA-DR ^lo/-^ CD11b^+^ CD14^-^ CD15^-^CD33^+^ [3]. In addition, novel phenotypic markers of MDSCs have emerged in different contexts and environments, with M-MDSC and PMN-MDSC phenotypes of CD11b^+^ Ly6G^-^ Ly6C^hi^ CD84^+^ and CD11b^+^ Ly6G^+^ CD84^+^ in mice, respectively. In humans, the phenotype of M-MDSC is CD14^+^/CD66b^-^ CXCR1^+^ or CD14^+^/CD66b^-^ CD84^+^ , while that of PMN-MDSC is CD15^+^ /CD66b^+^ CD14^-^ LOX1^+^ or CD15^+^/ CD66b^+^ CD14^-^CD84^+^ [24]. Interestingly, Je-In Youn et al. uncovered that M-MDSCs could acquire the phenotypic, morphological, and functional characteristics of PMN-MDSCs through histone deacetylase 2 (HDAC-2)-mediated epigenetic modifications and transcriptional silencing of the retinoblastoma (Rb) gene [25]. With the in-depth study of the origin and function of MDSCs, the phenotypic markers of MDSCs are continually being updated and developed, and the distinction between MDSCs may be more detailed and clear in the future.

### Amplification and activation of MDSCs

Albeit MDSCs are morphologically and phenotypically similar to neutrophils and monocytes, their activation processes and signaling are distinct from those of neutrophils and monocytes. Classical myeloid activation is generated in response to pathogen invasion and tissue injury and is predominantly induced in the form of pathogen-associated molecular patterns (PAMP), danger-associated molecular patterns (DAMP), and Toll-like receptors (TLR) [26], resulting in the rapid mobilization of monocytes and neutrophils in the bone marrow, respiratory burst, markedly enhanced phagocytosis, and production of large amounts of pro-inflammatory cytokines [27, 28]. However, this response is primarily aimed at eliminating foreign dangers and is of short duration. In contrast, pathological activation of myeloid cells occurs in the presence of chronic infections, cancers, autoimmune diseases, and persistent inflammatory environments. The first group of signals is generated in conditions such as infection, cancer, and inflammatory environments and includes granulocyte-macrophage colony-stimulating factor (GM-CSF), macrophage colony-stimulating factor (M-CSF), granulocyte colony-stimulating factor (G-CSF), vascular endothelial growth factor (VEGF), and prostaglandin E2 (PGE2) [5, 29, 30]. Under the regulation of transcriptional factors and regulators such as signal transducer and activator of transcription (STAT)-3, STAT5, interferon regulatory factor 8 (IRF8), and C/EBP-β [6], immature myeloid cells (IMCs) expand in large numbers, laying the quantitative foundation for the formation of a large number of MDSCs. Afterward, the second set of signals is generated by inflammatory cytokines and DAMP, including interleukin (IL)-1β, IL-4, IL-6, interferon (IFN)-γ, and tumor necrosis factor (TNF) [31]. The endoplasmic reticulum stress response has been recently discovered to promote the pathological activation of MDSCs [32]. At this stage, mononuclear/dendritic progenitor cells (MDP) and myeloblasts (MB) are transformed into pathologically activated MDSCs. MDSCs formed under these pathological conditions differ from the gene expression profile of mature bone marrow cells in healthy humans, thus conferring their immunosuppressive capacity, the main marker to distinguish the two.

### Recruitment of MDSCs

**Fig. 1:**
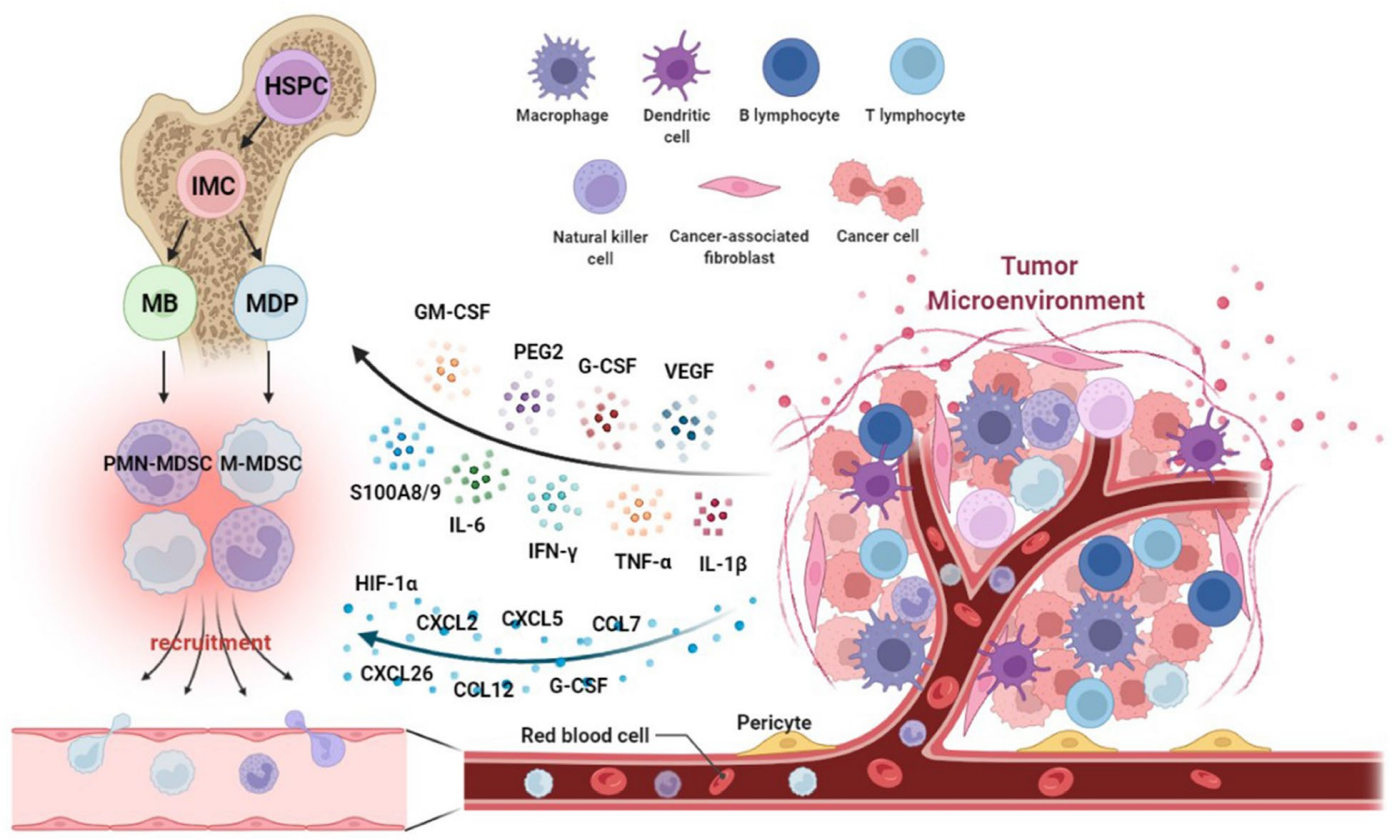
Schematic diagram of tumor microenvironment-mediated amplification, activation and recruitment of MDSCs. Figures were created in BioRender.com. *HSPC* hematopoietic stem progenitor cells, *IMC* immature myeloid cells, *MDP* mononuclear/dendritic progenitor cells, *MB* myeloblasts.

MDSCs can be recruited to the primary and metastatic tumor sites by chemokines released by the tumor. In ovarian cancer cells, the transcription factor Snail promotes the recruitment of MDSCs by upregulating the expression of the CXCR2 ligand CXCL2 through the NF-κB pathway [33]. CXCL2 has also been demonstrated to recruit MDSCs and promote tumor growth in breast cancer, pancreatic ductal adenocarcinoma, oral cancer, and glioblastoma, whereas knockdown of CXCR2 in MDSCs and knockdown of CCL2 reduced the recruitment of MDSCs to tumor sites in mice [34–37]. In renal cell carcinoma, intra-tumor levels of CXCL5 and CXCL8 were significantly correlated with the degree of MDSC infiltration [38]. In hepatocellular carcinoma, M-MDSCs are recruited through CXCL10 and TLR4 and promote tumor recurrence following liver transplantation [39]. In addition, various factors accumulated in the tumor microenvironment contribute to the recruitment of MDSCs. In a hepatocellular carcinoma murine model, the hypoxic environment in the tumor caused hypoxia-inducible factor-1 (HIF-1) to activate CXCL26 transcription in cancer cells, thereby recruiting MDSCs expressing the CXCL26 receptor CX3CR1 into the primary tumor site [40]. Moreover, tumor cells can synthesize indoleamine 2,3-dioxygenase (IDO) and rely on regulatory T cells (Treg) to deplete the essential amino acid tryptophan and recruit MDSCs [41]. Lastly, other chemokines such as CCL7 [42], CXCL4 [43], and CCL12 [44] have also been shown to mediate the recruitment of MDSCs in the tumor microenvironment. (Figure 1)

### The immunosuppressive function of MDSCs

Suppressing the immune response capacity of immune cells is a fundamental feature of MDSCs, which can exert immunosuppressive effects mainly through the following mechanisms.

1. Regulation of cellular metabolites: L-arginine is a key substance for T cell proliferation, and MDSCs exhibit high expression levels of inducible nitric oxide synthase (iNOS) and arginase-1 (ARG-1), which can degrade L-arginine, thus inhibiting the proliferation of activated T cells and reducing the expression of the TCR-ζ chain [8]. Besides, increased expression of ARG-1 in MDSCs can stimulate the production of extracellular matrix components, thereby promoting tissue remodeling and tumor growth [45]. Interestingly, it has recently been uncovered that soluble factors such as ARG-1 merely play a minor role in inhibiting T cell proliferation, and MDSCs are required to inhibit T cell proliferation through direct intercellular contacts [46]. Therefore, further studies are warranted to elucidate the role of ARG-1 in the immunosuppressive process of MDSCs. A recent study found that accumulation of the dicarbonyl radical methylglyoxal led to the metabolic phenotype of MDSCs and MDSC-mediated exhaustion of CD8^+^ T cells [47]. Meanwhile, the increased uptake and consumption of glucose by M-MDSC in the tumor microenvironment compromises immune cell metabolism and thus enables the immune escape of tumor cells [48]. Tumor cell glycolysis promotes liver-enriched activator protein (LAP) expression via AMP-activated protein kinase (AMPK)-ULK1 and autophagic pathways that mediate G-CSF and GM-CSF production to promote the immunosuppressive effects of MDSCs [49].GM-CSF also activates the transcription factor STAT5 in PMN-MDSCs to increase the expression of fatty acid transporter 2 (FATP2), which mediates the uptake of arachidonic acid (AA) and the synthesis of PGE2 to exert its immunosuppressive effects [50]. In addition, many lipid metabolic pathways are also involved in the immunosuppression of MDSC, as reviewed in [51].
2. Generation of reactive oxygen species (ROS) and nitric oxide (NO): MDSCs produce ROS that has a direct toxic effect on immune cells [52], while high levels of ROS also stimulate the expression of VEGF receptors on MDSCs, further contributing to their expansion and recruitment [53]. Additionally, the clearance of ROS leads to the differentiation of IMC isolated from tumor-bearing mice into DCs and macrophages in vitro, indicating that ROS could maintain the status of MDSCs [54]. ROS also induces TIPE2 (tumor necrosis factor-α–induced protein 8-like 2) to increase the expression of the pro-tumor mediator CCAAT/enhancer-binding protein-β, which regulates MDSC polarization [55]. In addition to ROS production, MDSCs also generate high levels of NO through the activation of iNOS and NO strongly induces the expression of cyclooxygenase 2 (COX-2) and HIF-1α [56], which is involved in PGE2 synthesis. This further upregulates the expression of IDO, IL-10, ARG-1, and other immunosuppressive markers [57], thus enhancing the suppressive effect of MDSCs on immune cells.
3. Induction of other immunosuppressive cells: M-MDSC exerted direct immunosuppressive effects on effector T cells and triggered Foxp3^+^ regulatory T cell (Treg) production by secreting TGF-β and IL-10 in a tumor mouse model, while TGF-β also induced the expansion of M-MDSCs [9, 58]. Recently, it has been reported that MDSCs can convert normal B cells into a unique population of programmed death receptor-1 negative, programmed death ligand-1 positive (PD-1^-^ PD-L1^+^) regulatory B cells (PD-1^-^ PD-L1^+^ Breg) in breast cancer and that this population has a stronger suppressive effect on T cell immune responses [59]. Furthermore, MDSCs can convert macrophages to an M2-like phenotype with immunosuppressive features, thereby promoting tumor growth [60].
4. Expression of negative immune checkpoint molecules: in patients with non-small cell lung cancer, MDSCs interact with T-cell immunoglobulin mucin 3 (TIM3) on T cells through the expression of Galectin-9 (Gal-9), which impairs the cytotoxic effect of CD8^+^ T cells and is closely associated with patient resistance to PD-1 monoclonal antibodies [61]. Tumor-derived MDSCs activate the phosphatidylinositol 3-kinase (PI3K)/protein kinase B (AKT)/nuclear factor kappa B (NF-κB) signaling pathway in PD-1^-^ PD-L1^+^ Bregs via the PD-1/PD-L1 axis, which mediates their immunosuppressive function [62]. MDSCs have also been found to express PD-1, PD-L1, and T-cell immunoglobulin and ITIM structural domain protein (TIGIT) ligands in human glioma tissues, thereby blocking TIGIT/PD1 to restore T-cell proliferation and immune function [63].

### MDSCs in hematologic malignancies

MDSCs have received widespread attention from clinicians and scientists owing to their role in suppressing immunity and stimulating tumor development. A large number of studies have now established that the number of MDSCs in the peripheral blood, bone marrow, and tumor infiltration sites is positively correlated with high tumor load, tumor stage, and poor prognosis. MDSCs are considered prognostic markers for hematologic malignancies as well, but do they only have detrimental effects in hematologic malignancies?

### Lymphoma

Serafini et al. first identified a population of cells expressing Gr1, F4/80, and IL-4Rα, low in MHC class I and II molecules and high in CD11b in a murine A20 B-cell lymphoma model, and further studies validated that this population of cells was MDSCs. Notably, this population of cells inhibited CD8^+^ T-cell proliferation and induced the recruitment and expansion of Tregs by ARG [64]. In a 2014 study, lenalidomide was reported to promote tumor regression and improve immunosuppressive status by reducing the frequency of MDSCs in A20 lymphoma mice; however, the exact mechanism has not been elucidated [65]. In another study conducted in 2016, Abedi-Valugerdi et al. observed a large infiltration of MDSCs in the spleen of EL4-luc2 lymphoma model mice as well as an elevated number of blood neutrophils, implying that tumors alter normal myelopoiesis and stimulate the production of MDSCs during tumor development [66]. In 2017, Zhen Xu et al. described that B-cell lymphoma model mice manifested an increased expression level of microRNA (miR)-30a in MDSCs. miR-30a activates the JAK2/STAT3 signaling pathway by decreasing the expression of suppressor of cytokine signaling-3 (SOCS3), thereby promoting MDSC differentiation and increasing the expression of their major immunosuppressive factors ARG-1, IL-10, and ROS [67]. In 2020, Lu Fei et al. noted an increased expression of PD-L1 and a significantly higher frequency of MDSCs in tumor tissues of patients with diffuse large B-cell lymphoma (DLBCL), which was evidently correlated with the immunosuppressive state of the patients. They used the NLRP3 inhibitor MCC950 to intervene in A20 B-cell lymphoma model mice and found that the proportion of MDSCs and other immunosuppressive cells (e.g., Treg) in the tumor tissue and spleen of the mice declined after treatment. However, the proportion of tumor-infiltrating MDSCs was increased after combined anti-PD-L1 treatment, suggesting that blockade of PD-L1 could affect the immune recovery function of MCC950 [68]. A study identified calmodulin kinase kinase 2 (Camkk2) as a target for the accumulation of MDSCs in E.G7-OVA tumor-bearing mice. Indeed, knockdown of Camkk2 in mice resulted in an enhanced anti-tumor immune response of T cells and reduced accumulation of MDSCs, slowing down the growth of tumor cells. In contrast, the ability to restore tumor growth was restored by the overtransplantation of MDSCs into Camkk2^-/-^ mice, suggesting the critical role of MDSCs in the tumor immunosuppressive process and a possible role of Camkk2 as a target to inhibit MDSC expansion [69].

In clinical terms, Romano et al. found an increased proportion of M-MDSC, PMN-MDSC, and CD34^+^ MDSC subpopulations in the peripheral blood of 60 newly diagnosed Hodgkin's lymphoma (HL) patients compared to healthy controls. The number of MDSCs was lower in patients who achieved CR compared to those who did not achieve complete remission (CR) after chemotherapy [70]. In contrast, Amini RM et al. reported a higher number of PMN-MDSCs in the peripheral blood of 19 HL patients compared to healthy controls, while there was no significant difference in M-MDSCs [71], likely due to differences in the number of patients in the study, the stage of the disease and differences in phenotypic markers for MDSCs. Similarly, Marini et al. found a higher proportion of PMN-MDSCs (CD66b^+^ CD33^dim^ HLA-DR^-^) in 124 patients with B-cell lymphoma (both HL and B-NHL) and that this was correlated with international prognostic index and disease status, while depletion of CD66b^+^ MDSCs restored T-cell proliferation [72]. A significant increase in the number of MDSCs in the peripheral blood of DLBCL patients has also been reported, but only the number of M-MDSCs correlated with the international prognostic index, event-free survival, and the number of circulating Tregs, and IL-10, S100A12, and PD-L1, which are associated with the immunosuppressive effects of MDSCs, were expressed at an increased level in DLBCL patients, while inhibition of these molecules could increase T cell proliferation [73]. According to a recent study, the number of M-MDSCs in newly diagnosed and relapsed DLBCL patients was positively correlated with tumor progression and negatively correlated with overall survival (OS), while IL-35 mediated the accumulation of M-MDSCs in DLBCL patients and anti-IL-35 treatment significantly reduced M-MDSC levels in a Ly8 DLBCL murine model [74]. Furthermore, a clinical trial using lenalidomide combined with R-GDP (rituximab plus gemcitabine, cisplatin, and dexamethasone) for relapsed/refractory DLBCL demonstrated that both MDSCs and Tregs were elevated in circulating numbers in DLBCL patients, were reduced and approached healthy control levels in patients with overall survival > 24 months after treatment, and determined that vitamin D-deficient DLBCL patients had higher levels of MDSCs and Tregs; these results signal that vitamin D supplementation may result in enhanced treatment outcomes in these patients [75, 76]. In other recent studies, an increased number of MDSCs was also detected in DLBCL patients at diagnosis and was associated with a poorer disease prognosis [77, 78].

Furthermore, in patients with extranodal NK/T-cell lymphoma (ENKL), total MDSCs (CD33^+^ CD11b^+^ HLA-DR^-^) and M-MDSCs were independent prognostic factors for patient disease-free survival (DFS) and OS, and studies have also found that the inflammatory cytokine IL-17 produced by CD4^+^ Th17 cells in ENKL patients may promote the effect of MDSCs on the inhibition of T cell proliferation [79]. There are no literature reports on other types of NHL, such as set cell lymphoma or follicular lymphoma, associated with MDSCs.

### Multiple myeloma

Veirman's team discovered an accumulation of MDSCs in the bone marrow during the early stages of MM progression in a 5TMM mouse model, and MDSCs could appear in the peripheral blood at later stages of the disease. Moreover, soluble factors from MM cells could promote the survival of MDSCs by increasing the expression of the anti-apoptotic protein Mcl-1. In in vitro studies, bone marrow mesenchymal stem cell (BMSC)-derived exosomes could directly induce the survival of MM MDSCs and increase NO release from MDSCs by activating the STAT3 and STAT1 pathways and increasing the expression of anti-apoptotic proteins Bcl-xL and Mcl-1, thereby enhancing their suppressive function against T cells. Conversely, MDSCs also promoted MM cell survival via Mcl-1 and Bcl-2 and contributed to their resistance to bortezomib and melphalan [80–82]. Another study also established that co-culture of MM-derived but not healthy donor-derived MSCs with PMN-MDSCs resulted in PMN-MDSCs with immunosuppressive functions [83]. Besides, the presence of S100A9 and its receptor TLR4 was detected in MDSCs of MM model mice, and the former served as a chemoattractant for MM cells to trigger MDSCs to express and secrete inflammatory factors such as TNF-α, IL-6, and IL-10. Blocking S100A9 did not directly influence MDSC accumulation but rather decreased the expression of inflammatory cytokines in MDSCs [84]. Furthermore, MM-derived Galectin-1 mediates the pro-tumorigenic effects of M-MDSCs by interacting with CD304 on M-MDSCs and facilitates MM progression following autologous stem cell transplantation (ASCT) [85]. These results suggest that in the MM microenvironment, various cells and factors interact with each other and together influence the development of MM.

In humans, Brimnes et al. first reported an increased number of M-MDSCs in the peripheral blood of MM patients compared to healthy controls in 2010 [86]. Later, Wang et al. showed that M-MDSC levels were positively correlated with MM recurrence and negatively correlated with treatment outcomes [87]. Meanwhile, a study found that bone marrow neutrophils from MM patients exhibited MDSC activity [88], and further immunogenomic identification suggested that CD11b^+^ CD13^+^ CD16^+^ neutrophils in MM are G-MDSCs [89]. G-MDSCs have been reported to be highly accumulated in the bone marrow and peripheral blood of MM patients compared to healthy donors, and this abundance has also been positively correlated with disease activity [83, 90, 91]. In terms of treatment, there are conflicting reports on the impact of the proteasome inhibitor bortezomib, the immunomodulatory agent lenalidomide (LEN), and DC vaccination on MDSCs in the treatment of MM (reviewed in [92]). The immunomodulatory drugs LEN and pomalidomide reduce CCL5 and macrophage migration inhibitory factor (MIF) expression in myeloma cells, thereby suppressing the generation of MDSCs [93]. The combined use of LEN and PD-1 monoclonal antibodies can further curtail the number of MDSCs [94]. In addition, large numbers of M-MDSCs can reduce the cytotoxicity of pre-ASCT melphalan and are associated with a poorer clinical prognosis [95]. A recent study illustrated that the demethylating agent decitabine (DAC) inhibited MM cell proliferation and enhanced the immune response of autologous T cells by depleting M-MDSCs [96]. The CD38 antibody daratumumab also improved the antitumor immunity of MM patients by diminishing the number of immunosuppressive cells such as Tregs, Bregs, and MDSCs [97]. In addition, estrogen has been associated with promoting MM progression by enhancing the immunosuppressive function of bone marrow MDSCs [98]. High levels of IL-18 in the MM bone marrow microenvironment increase the immunosuppressive capacity of MDSCs via C/EBPβ, and elevated IL-18 levels in the bone marrow of MM patients are consequently associated with poor patient prognosis [99]. MIF in the MM microenvironment induces CD84 expression in bone marrow cells, leads to elevated expression of genes related to differentiation of MSDCs, and upregulates PD-L1 expression on MDSCs, thereby suppressing T-cell function [100]. These newly identified molecules are anticipated to be utilized to target MDSCs in order to treat MM.

### Leukemia

In acute leukemia, a study using the leukemic cell line TIB-49 implanted in mice resulted in the expansion and accumulation of MDSCs in the bone marrow and spleen, and the oncoprotein MUC1 was found to be a key driver of extracellular vesicle (EV)-mediated expansion of MDSCs [101]. Additionally, Tohumeken et al. determined that palmitoylated proteins on acute myeloid leukemia (AML)-derived EVs promote the differentiation of monocytes toward MDSCs through TLR2/Akt/mTOR signaling [102]. Sun et al. found a significant increase in MDSCs (CD33^+^ CD11b^+^ HLA-DR^lo/-^) in the bone marrow of adult AML patients with high minimal residual disease (MRD). Indeed, the patients had significantly higher levels of bone marrow MDSCs than those in the medium and low MRD groups [103]. In another instance, higher amounts of M-MDSCs and e-MDSCs were identified in the peripheral blood of AML patients [104], and the elevated circulating MDSCs were significantly correlated with lower CR and higher relapse/refractory rates and lower long-term survival in these patients [105]. It has been found that intervention of AML cells with the AML chemotherapeutic drug agranulocyte (Ara-C) increases TNF-α production, which in turn activates the IL-6/STAT3 and NF-κB pathways to amplify MDSCs and enhance their immunosuppressive function [106]. Hwang et al. demonstrated that the combination of Ara-C, the CXCR4 inhibitor Plerixafor, and a PD-L1 monoclonal antibody in an AML murine model resulted in a decrease in the number of Tregs and MDSCs in the peripheral blood as well as in bone marrow leukemic cells, inferring that the treatment of AML by modulating the leukemic microenvironment is a very promising strategy [107]. In addition, the number of MDSCs was significantly increased in the peripheral blood of patients with B-cell acute lymphoblastic leukemia (B-ALL) [108]. Hotari et al. noted a decrease in M1-type macrophages and effector T cells and an increase in M2-type macrophages and MDSCs in bone marrow samples of ALL patients compared to healthy controls, and that negative immune checkpoint molecules of MDSCs (such as PD-1 and CTLA-4) expression were increased in association with immunosuppression [109]. In patients with acute promyelocytic leukemia (APL), tumor-activated intrinsic lymphocytes (ILC2) secrete IL-13 to induce M-MDSC production and support tumor growth, whereas all-trans retinoic acid (ATRA) treatment reverses the ILC2-induced increase in MDSCs [110].

In chronic granulocytic leukemia (CML), MDSCs and the levels of their immunosuppressive markers IL-10 and ARG1 are elevated, and the tyrosine kinase inhibitor (TKI) imatinib and dasatinib treatments can reduce MDSC levels to within the normal range [111, 112]. Further studies established that TKI treatment reduced the proportion of G-MDSCs, but only patients treated with dasatinib had a significant reduction in the number of M-MDSCs. Therefore, M-MDSCs may act as a prognostic factor in CML patients treated with dasatinib [113]. In a study regarding chronic lymphocytic leukemia (CLL), elevated M-MDSC levels were observed in the peripheral blood of 50 newly diagnosed patients and were associated with poorer survival [114]. Tregs and MDSCs can revert to normal levels in CLL patients within 1–2 years after ibrutinib treatment [115]. Through further studies, PMN-MDSCs were found to have a greater impact on immunosuppression than M-MDSCs in CLL patients, while ibrutinib further lowered the number of PMN-MDSCs and altered the differentiation of MDSCs, inducing naïve T cells toward Th1 cells and away from Th2 cells, thus improving the tumor microenvironment in leukemia [116].

### Myelodysplastic syndromes

In myelodysplastic syndromes (MDS), MDSCs suppress the immune response of T cells by inducing Treg proliferation in the MDS bone marrow microenvironment, preventing T cells from clearing malignant clonal cells [117]. Meanwhile, the high expression of PD-L1 in MDSCs triggers hematopoietic cell death by binding to PD-1 of hematopoietic stem progenitor cells (HSPCs), resulting in ineffective bone marrow hematopoiesis [118]. Furthermore, MDSCs can also secrete large amounts of S100A9 in MDS, which on the one hand, can interact with TLR-4 on HSPCs to activate downstream inflammatory signaling pathways, and on the other hand, bind to their cellular surface CD33 to trigger the production of immunosuppressive cytokines IL-10 and TGF-β to directly inhibit hematopoiesis through their immune receptor tyrosine-based inhibitory motifs [119]. Our previous also study identified that Gal-9, a ligand of the immune checkpoint molecule TIM3 highly expressed in the MDSCs of MDS patients, can bind to the TIM3 that is highly expressed on CD8^+^ T cells to suppress their immune function, leading to CD8^+^ T cell exhaustion [120]. It was also established that MDSCs can govern the expression of the immunosuppressive molecule ARG1 through the STAT3 pathway, which in turn impacts the antitumor immune response of CD8^+^ T cells [121]. Hence, exploring the role of negative immune checkpoint molecules highly expressed by MDSCs in MDS patients will aid in exploring approaches to suppress MDSCs for the treatment of MDS.

Since most malignant hematologic diseases originate from malignant clonal proliferation of bone marrow cells, whether MDSCs originate from the same lineage or population as malignant clonal cells necessitate further in-depth investigations. Many drugs or targeted therapies for hematologic tumors have been found to inhibit MDSCs as well, raising the question of whether these drugs have common or similar therapeutic mechanisms for tumor cells and MDSCs. Moreover, recent studies have also established that epigenetic and metabolic regulation are closely related to the generation of immunosuppressive functions in MDSCs, and hence the combination of epigenetic-related therapeutic agents (e.g., azacitidine and chidamide) with traditional chemotherapeutic agents or novel drugs for the treatment of malignant hematological diseases may yield superior therapeutic outcomes.

### MDSCs in hematopoietic stem cell transplantation

In the tumor microenvironment of the hematologic malignancies described above, MDSCs mostly play the role of “villains”, suppressing the anti-tumor immune response of immune cells and disrupting the immune microenvironment, thus promoting tumorigenesis and progression. In HSCT for hematologic malignancies, the relationship between GVL effects, graft-versus-host disease (GVHD), and MDSCs is more intricate.

MDSCs have been detected in peripheral blood hematopoietic stem cells (G-PBSC) after mobilization with G-CSF [122]. A multicenter study determined that bone marrow hematopoietic stem cell (G-BM) transplantation after G-CSF mobilization enhanced relapse-free survival with a lower incidence of GVHD compared to G-PBSC transplantation, which was associated with a higher number of MDSCs in the grafts [123]. A real-world study also found that allo-HSCT mobilized with pegylated granulocyte colony-stimulating factor alleviates severe acute graft-versus-host disease by enriching M-MDSCs in the graft and is a feasible and safe treatment modality [124]. Using transcriptome sequencing, Andrea et al. identified a substantial upregulation of genes that promote DNA replication, cell cycle, and cell division in G-CSF mobilized PMN-MDSCs, such as the marker of cell proliferation Ki-67 (MKI67), topoisomerase II alpha (TOP2A) and cyclin B (CCNB2) [125]. This study revealed the underlying mechanism for the marked increase in the number of MDSCs after G-CSF mobilization. Encouragingly, a retrospective study discovered an improved anti-leukemic effect of DLI after administration of G-CSF compared to conventional donor lymphocyte infusion (DLI) in patients who relapsed after allo-HCT, given that it was enriched in MDSCs and did not increase the cumulative GVHD incidence [126]. Furthermore, umbilical cord mesenchymal stem cells promote MDSCs enrichment and prevent GVHD after HSCT by secreting CXCL1 [127] and HLA-G [128]. Zhang et al. investigated the inhibition of GVHD by MDSCs while preserving the GVL effect and noted that MDSCs induced NKG2D expression on T cells and simultaneously suppressed GVHD by upregulating Tregs [129]. Delayed recovery of M-MDSC and invariant natural killer T cell (iNKT) numbers after transplantation was related to an increased incidence of grade III–IV acute GVHD, but the combination of lower levels of M-MDSCs and higher levels of iNKT cells was associated with enhanced GVL effects and reduced leukemic relapse [130]. This demonstrates that MDSCs need to be balanced with other types of immune cells in order to play a positive regulatory role in HSCT.AUTHOR: As References [23] and [134] are same, we have deleted the duplicate reference and renumbered accordingly. Please check and confirm.correct

Furthermore, a major obstacle facing the field of GVHD mitigation by transfusion of immunosuppressive cells is the disruption of the immune system, the delayed growth of immune cells, and the risk of infection. Pretreatment regimens combined with stem cell infusion and the subsequent occurrence of tissue damage create an inflammatory environment that may lead to excessive M-MDSC expansion, in which case increased M-MDSC levels predict higher non-relapse mortality [131]. In contrast, it is challenging to extract large amounts of MDSCs from humans in a short period of time owing to their low numbers in humans, which may take too long for their use as a treatment for severe acute GVHD, and the ideal method for in vitro culture expansion of MDSCs has not been determined so far [132]. Interestingly, recent studies have found that the use of the immunosuppressant cyclophosphamide following allo-HSCT resulted in early recovery of MDSCs and a reduction in the incidence of GVHD [23] and that cyclosporine A, another drug, inhibited the opening of the mitochondrial permeability transition pore (MPTP) in PMN-MDSCs, thereby reducing MDSC damage in the inflammatory environment of GVHD [133].

As is well documented, to maintain the GVL effect and prevent and treat GVHD after HSCT, the timing, dose, and discontinuation of immunosuppressive drug interventions are critical. According to some studies, immunosuppressive drugs can enhance the function of MDSCs and hence anti-GVHD. However, MDSCs also exert a pro-tumor effect, and therefore further studies are required to confirm whether MDSC levels should be regularly monitored in vivo and whether MDSCs can cause MRD or tumor recurrence.

### Targeting MDSCs

MDSCs play diverse roles in hematologic malignancies and HSCT. Inhibiting the differentiation of MDSCs in the bone marrow microenvironment, minimizing recruitment, and removing MDSCs from the microenvironment may be effective approaches in targeting MDSCs to treat the disease, and numerous therapeutic drugs for hematologic diseases have also been found to be effective in influencing MDSCs (Table 1). That being said, the development of MDSC-associated cell therapies for the prevention and treatment of GVHD following HSCT is a very promising direction.

**Table 1:** Studies targeting MDSCs in malignant hematologic diseases and HSCT

Therapeutic drugs	Targeting process	Disease	Action effect	References
Daratumumab	Reduction in the number of MDSCs	MM	Reduces the number of CD38^+^ MDSCs.	[97]
All-trans retinoic acid	Inhibition of M-MDSC production	APL	Induces APL primitive cell differentiation and death and inhibits PGD2/ILC2/IL-13 axis-induced MDSC generation.	[110]
Imatinib/dasatinib	Reduction in the number and immunosuppressive effect of MDSCs	CML	Reduces the number of MDSCs and their ARG-1, MPO, and IL-10 levels.	[111]
Ibrutinib	Reduction in the number of MDSCs	CLL	Reduces MDSC numbers and alters the differentiation of MDSCs, inducing naïve T cells towards Th1 cells and away from Th2 cells.	[116]
Tadalafil	Inhibition of immunosuppressive effects of MDSCs	R/R MM	Reduces the levels of ROS, ARG-1, and iNOS in MDSCs and restores the anti-tumor immune response of T cells.	[134]
Bisphosphonates	Reduction in the number of MDSCs	MM	Reduces the number of MDSCs and decreases their ability to differentiate into osteoblasts.	[153]
Bendamustine	Enhancement of immunosuppressive function of MDSCs	HSCT	Enhances immunosuppression in MDSCs and reduces GVHD.	[154]

*APL* acute promyelocytic leukemia, *R/R MM* relapsed/refractory multiple myeloma, *CML* chronic myeloid leukemia, *CLL* chronic lymphocytic leukemia, *PGD2* prostaglandin D2, *ILC2* Group 2 innate lymphoid cells, *ARG*-1 arginase 1, *ROS* reactive oxygen species, *MPO* myeloperoxidase, *HSCT* hematopoietic stem cell transplantation, *GVHD* graft-versus-host disease

### Inhibiting the differentiation and function of MDSCs

Blocking the differentiation of other cells to MDSCs and attenuating the immunosuppressive function of MDSCs are also valid methods of targeting MDSCs. ATRA is a well-known inducer of differentiation, and a study reported that it reversed ILC2-induced MDSC generation in APL [110]. Noonan et al. demonstrated that the phosphodiesterase 5 (PDE5) inhibitor tadalafil reduced the expression of ROS, ARG-1, and iNOS in the MDSCs of MM patients and restored the antitumor immune response of T cells [134]. In addition, Grauers et al. utilized histamine hydrochloride (HDC) to inhibit NOX2, lower ROS production in MDSCs, and impair tumor growth in lymphoma mice. Moreover, a subsequent phase IV clinical trial (NCT01347996) further demonstrated a significant reduction in M-MDSCs in AML patients treated with HDC and low-dose IL-2, resulting in favorable therapeutic outcomes [135]. In addition, pharmacological blockade of FATP2 expression in MDSCs reduced lipid accumulation, decreased ROS levels, attenuated the immunosuppressive activity of MDSCs, and reduced PD-L1 expression on immune cells, thereby enhancing the effect of tumor immunotherapy [136]. A recent study found that metabolic reprogramming of the immunosurveillance-activating nanodrug-assembled doxorubicin (MRIAN-Dox) inhibited M2-type pyruvate kinase (PKM2) activity and reduced ROS levels in MDSCs in a T-cell acute lymphoblastic leukemia (T-ALL) mouse model, thereby interfering with their immunosuppressive function and increasing their differentiation to normal bone marrow cells [137].

### Reducing MDSC recruitment

Another therapeutic approach is to prevent the migration of MDSCs to tumor sites, and chemokine receptors play a critical role in recruiting MDSCs to tumor sites (as previously described). Clinical trials have been conducted in MM using the CXCR4 antibody Ulocuplumab combined with LEN or bortezomib plus dexamethasone and have shown satisfactory efficacy [138]. Colony-stimulating factor receptor (CSF-1R) is essential for the survival and recruitment of MDSCs, and treatment with CSF-1R inhibitors has shown promising results. Kumar et al. exposed that inhibition of CSF-1R and CXCR2 reduced G-MDSC levels and improved efficacy against PD-1 antibodies in a mouse lymphoma model [139]. Tyner et al. employed the CSF-1R inhibitor GW-2580 to reduce CD33^+^ MDSC recruitment in the bone marrow of AML patients [140]. Likewise, imatinib targets GSF1R to reduce the number of circulating G-MDSCs in CML patients [113]. Lu et al. supplemented epigenetic treatment with a low-dose DNA methyltransferase inhibitor (5-azacytidine) and a histone deacetylase inhibitor (entinostat) in a mouse model of lung metastasis, which disrupts the formation of the pre-metastatic microenvironment by suppressing the migration of MDSCs through the downregulation of CCR2 and CXCR2, and by promoting MDSC differentiation into an interstitial macrophage-like phenotype [141]. Entinostat also inhibits the VEGF, ErbB, and mTOR pathways in PMN-MDSCs, thereby suppressing STAT3 activity and consequently reducing Arg-1, iNOS, and COX2 activity [142]. In addition, HDAC inhibitors upregulate PD-1 or PD-L1 expression on tumor or immune cells, sensitizing tumor-bearing mice to anti-PD-1/PD-L1 antibodies [143].

### Depletion of MDSCs

Qin et al. were able to deplete MDSCs in blood, spleen, and tumor and slow tumor growth in vivo in various lymphoma model mice (A20, EG7, EL4) by screening candidate peptides that specifically bind MDSCs and synthesizing peptide-Fc fusion proteins (peptidomes), which were administered intravenously without influencing other immune cells [144]. More importantly, Fultang et al. uncovered that a CD33 monoclonal antibody (Gemtuzumab) increased the death of CD33^+^ MDSCs, restored T cell proliferation, and showed satisfactory results in subsequent clinical trials [145–147]. Cheng et al. developed a CD33/CD3 bispecific T cell splice agent, AMV564, which effectively depleted CD33^hi^ MDSCs in MDS and improved T-cell antitumor activity, and its combination with immune checkpoint inhibitors improved patient resistance to AMV564 [148]. In addition, similar T cell binding bispecific antibodies in MM are capable of redirecting host T cell cytotoxicity to malignant clonal MM cells as well as MDSCs in an MHC-independent manner [149]. Masoud F et al. found that activation of LXR/ApoE/LRP8 inhibited MDSC survival. Besides, in a human dose-escalation phase 1 trial (NCT02922764), an LXR agonist (RGX-104) reduced MDSC abundance in patients. Lastly, LXR/ApoE activation therapy triggered antitumor immune responses in cytotoxic T lymphocytes and enhanced T cell activation in various immune-based therapies [150].

### MDSC implantation

In contrast to MDSCs in hematologic malignancies, MDSCs have been found to be beneficial in preventing the development of GVHD in HSCT and preserving the GVL effects of grafts [126, 129]. Wang et al. transplanted G-CSF-stimulated generated e-MDSCs into acute GVHD xenogeneic model mice and observed that these cells prevented the occurrence of GVHD and that e-MDSCs inhibited T cell proliferation in a TGF-β-dependent manner, modulated Th cell differentiation from Th1 to Th2 and promoted Treg production. Collectively, these effects facilitated the establishment of immune tolerance in HSCT [151]. Drujont et al. showed that repeated injections of MDSCs or a single injection of lipopolysaccharide-activated MDSCs in a skin graft model significantly prolonged the survival time of allografts [152], indicating a potential therapeutic strategy for the clinical application of MDSC transplantation. However, the timing and number of transplanted MDSCs need to be considered in the context of the patient's condition and the possibility of GVHD recurrence, and therefore, this approach is still being explored.AUTHOR: As References [5] and [31] are same, we have deleted the duplicate reference and renumbered accordingly. Please check and confirm.correct

## Conclusions

**Fig. 2:**
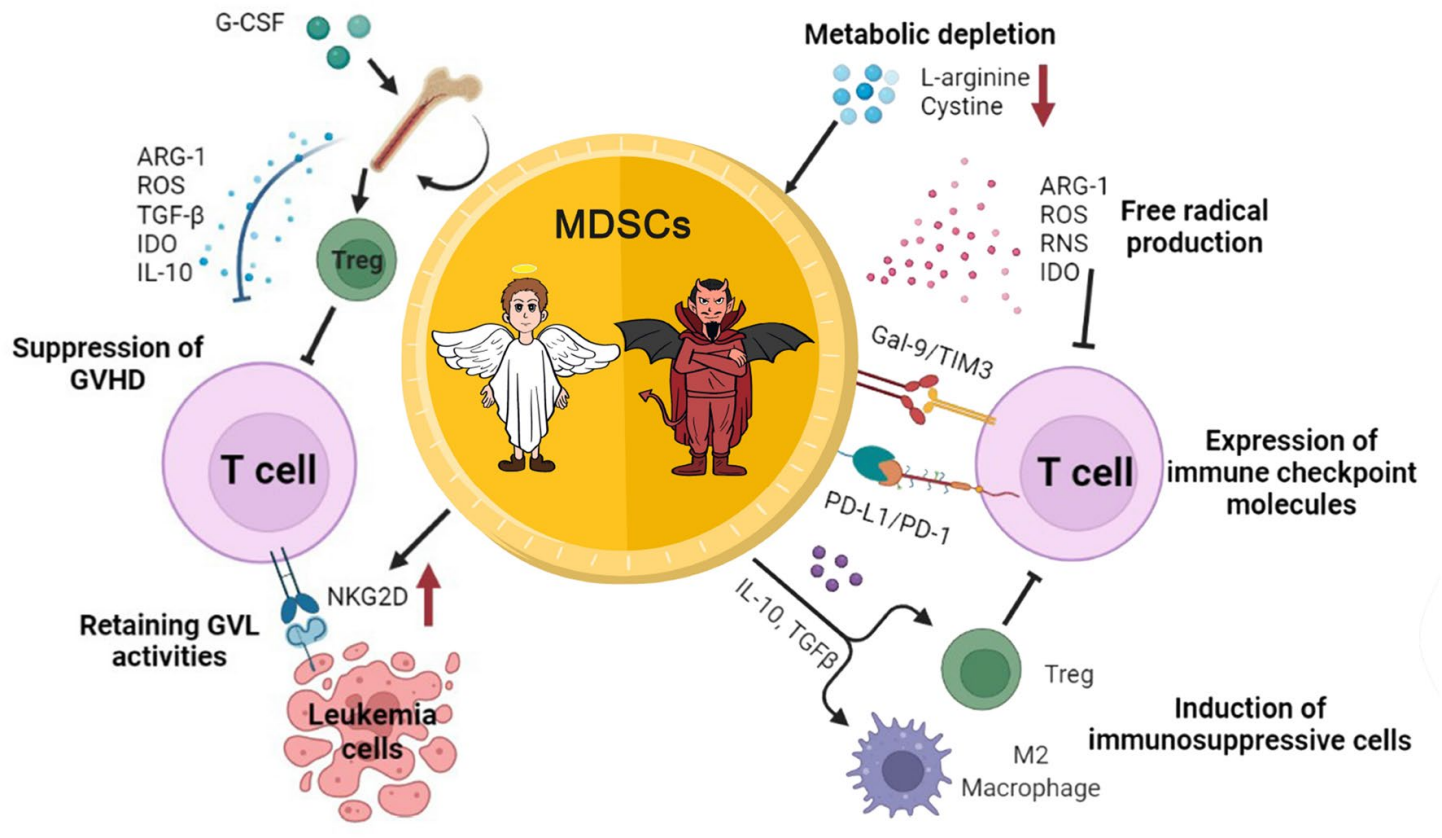
The different roles of MDSCs in hematologic malignancies and HSCT. Figures were created in BioRender.com.

Although MDSCs inhibit the anti-tumor capacity of immune cells and exhibit pro-tumor characteristics, they also offer new hope for the prevention or alleviation of GVHD after HSCT in patients with malignant hematological diseases (Figure 2). We now have a clear understanding of the origin and development of MDSCs, but their genomic and metabolic differentiation mechanisms remain elusive, and the intricate relationship between MDSCs and other cells in the tumor microenvironment warrants further investigation. Multi-omics technologies such as single-cell sequencing and spatial transcriptome may be an important approach to unravel the deeper mechanisms underlying the dual action of MDSCs in the future. Moreover, the treatment of malignant hematological diseases has entered the era of cellular immunotherapy, and MDSCs have been demonstrated to inhibit the anti-tumor capacity of CAR-T cells through their immunosuppressive activity [155, 156]. There are already ways to inhibit or remove MDSCs by altering their differentiation direction [157], pattern recognition receptor agonist delivery [158], and developing NK cells expressing chimeric activated receptors, thus enhancing the anti-tumor effects of CAR-T cells [159]. In the future, the construction of immune cells into CAR-T or CAR-NK cells with common targets against tumor cells and MDSCs may be an important direction.

## Data Availability

The datasets generated for this study are available on request to the corresponding author.

## Abbreviations

HSPC: Hematopoietic stem progenitor cells
IMC: Immature myeloid cells
MDP: Mononuclear/dendritic progenitor cells
MB: Myeloblasts
MDSC: Myeloid-derived suppressor cells
APL: Acute promyelocytic leukemia
MM: Multiple myeloma
CML: Chronic myeloid leukemia
CLL: Chronic lymphocytic leukemia
PGD2: Prostaglandin D2
ILC2: Group 2 innate lymphoid cells
ARG-1: Arginase 1
ROS: Reactive oxygen species
MPO: Myeloperoxidase
HSCT: Hematopoietic stem cell transplantation
GVHD: Graft-versus-host disease
GVL: Graft-versus-leukemia
